# Mountaineers on Mount Everest: Effects of age, sex, experience, and crowding on rates of success and death

**DOI:** 10.1371/journal.pone.0236919

**Published:** 2020-08-26

**Authors:** Raymond B. Huey, Cody Carroll, Richard Salisbury, Jane-Ling Wang

**Affiliations:** 1 Department of Biology, University of Washington, Seattle, Washington, United States of America; 2 Department of Statistics, University of California, Davis, California, United States of America; 3 Information Technology Division, University of Michigan, Ann Arbor, Michigan, United States of America; University of South Carolina, UNITED STATES

## Abstract

Mount Everest is an extreme environment for humans. Nevertheless, hundreds of mountaineers attempt to summit Everest each year. In a previous study we analyzed interview data for all climbers (2,211) making their first attempt on Everest during 1990–2005. Probabilities of summiting were similar for men and women, declined progressively for climbers about 40 and older, but were elevated for climbers with experience climbing in Nepal. Probabilities of dying were also similar for men and women, increased for climbers about 60 and older (especially for the few that had summited), and were independent of experience. Since 2005, many more climbers (3,620) have attempted Everest. Here our primary goal is to quantify recent patterns of success and death and to evaluate changes over time. Also, we investigate whether patterns relate to key socio-demographic covariates (age, sex, host country, prior experience). Recent climbers were more diverse both in gender (women = 14.6% vs. 9.1% for 1990–2005) and in age (climbers ≥ 40 = 54.1% vs. 38.7%). Strikingly, recent climbers of both sexes were almost twice as likely to summit–and slightly less likely to die–than were comparable climbers in the previous survey. Temporal shifts may reflect improved weather forecasting, installation of fixed ropes on much of the route, and accumulative logistic equipment and experience. We add two new analyses. The probability of dying from illness or non-traumas (e.g., high-altitude illness, hypothermia), relative to dying from falling or from ‘objective hazards’ (avalanche, rock or ice fall), increased marginally with age. Recent crowding during summit bids was four-fold greater than in the prior sample, but surprisingly crowding has no evident effect on success or death during summit bids. Our results inform prospective climbers as to their current odds of success and of death, as well as inform governments of Nepal and China of the safety consequences and economic impacts of periodically debated restrictions based on climber age and experience.

## Introduction

Nearly a century has passed since mountaineers began mounting full-scale attempts to climb Mount Everest (8850 m) in 1922 [1]. The first ascent was finally made in 1953 [2], and 4,346 have now reached the summit through spring 2019. Although Everest is no longer an exclusive achievement, many mountaineers still view Everest as the ultimate testing ground for high-elevation adventure. Many of their attempts and disasters have been chronicled and debated in books, movies, television, and other media. However, actual rates of success and of death–as well as whether those rates have shifted over time–have rarely been quantified (below); and those that have are sometimes calculated improperly [see 3]. Accurate statistical data inform not only prospective climbers debating whether to attempt this peak, but also governments debating whether to institute restrictions on climbers.

The scope of climbing–and its associated triumphs and tragedies–on Everest as well as on other Nepalese peaks can now be quantified accurately by accessing *The Himalayan Database* (https://www.himalayandatabase.com, “HD”), which is based on the archival interview records of the late Ms. Elizabeth Hawley [4]. HD covers all known attempts on 468 Nepalese and border peaks (including Mt. Everest) from 1905 through spring 2019. HD currently records detailed information on 10,363 expeditions, 60,162 climbers, and 28,587 high altitude porters. HD is updated twice each year and is freely available.

Several studies have used HD to evaluate mountaineering successes and its risks [5–10] (additional references are listed on the HD website). For example, we [11] previously analyzed relationships of age and of sex on rates of “success” (= reached summit), of “complete success” (summited and returned safely to base camp), and of death of mountaineers on Everest. That analysis, which focused on 2,211 climbers making their first attempt on Everest between 1990–2005, uncovered six key patterns:

1. Most climbers were men, but the proportion of women climbers had increased over prior time periods.
2. Men and women had similar odds of success, complete success, and dying.
3. The proportion of “old” climbers (≥ 60) had increased over prior time periods.
4. Climbers older than about 40 years had progressively lower rates of summiting; and climbers older than about 59 had marginally increased rates of dying, especially the few that had summited.
5. Probabilities of success and of complete success have improved in the recent period, while probabilities of death have declined slightly.
6. Prior experience climbing on a Nepalese or border peak was associated with an elevated success rate but was unrelated to death rate.

During recent years (2006 –spring 2019), 3,620 additional (first-time) climbers have attempted Everest during spring, such that the total number of attempts after 1989 is 2.5 times greater than in our previous study [11]. Moreover, the total number of attempts by climbers older than 59 has increased by 3.6-fold (56 to 200). Here we take advantage of these expanded data to take a fresh and updated look at mountaineering on Everest: we evaluate whether our findings for an earlier era are still supported, gain enhanced statistical power needed to re-evaluate the apparent increased risk faced by old climbers (above), and provide climbers, their families, and permit agencies with contemporary statistical patterns of mountaineering on Everest. Of course, retrospective and descriptive data do not permit assignment of causal factors behind these descriptive patterns; but we can nonetheless discuss several factors that may be involved.

## Materials and methods

### Data sources and exclusions

Mountaineering data analyzed here were amassed over five decades by Elizabeth Hawley (deceased 2018) but have been collected primarily by Billi Bierling and colleagues since 2016. Original written records were obtained via interviews in Kathmandu or correspondence and converted to *The Himalayan Database* by Richard Salisbury. For expeditions climbing in Nepal, records should be complete, as interview records are shared and compared with official permit lists at the Ministry of Tourism. Most expeditions climbing in China start and end in Kathmandu; and records for these should be complete. A few commercial expeditions start and end in Tibet, and records for these should be largely complete for member clients (see below).

We downloaded data for climbers attempting Everest between 1922 and spring 2019. For most analyses, however, we compared data for 1990–2005 versus 2006–2019. We excluded data from earlier years [‘exploratory’ and ‘expeditionary’ periods,7] because climbing techniques, equipment, and weather forecasts then were less developed than at present.

We compiled data for “members,” that is climbers formally listed on expedition climbing permits and who will attempt to summit. As in our prior study [11], we considered only members making their first attempt on Everest, thereby avoiding non-independence. Also, we excluded high-altitude porters and assistants (often called “Sherpas” or “hired”), who often were not attempting to summit, for whom age is sometimes unknown, and for whom records are incomplete for some expeditions in Tibet. Starting with a base of 14,537 member records (1921–2019 inclusive), we then progressively excluded 1,395 members who did not climb or intend to summit (“msmtterm” = 17 in HD), 18 members with special assignments (e.g., base camp leader, movie team, press director), 44 members on ski expeditions, 24 members attempting to traverse the summit (i.e., from Tibet to Nepal or vice versa), and 358 members having unknown ages. This left 12,698 attempts.

For our primary subset we further deleted 2,108 members who climbed prior to 1990, 3232 members who were not first-time climbers on Everest, 826 members who climbed in seasons other than spring [12], and 677 members who used non-commercial routes [i.e., all routes other than these (Nepal: S Col-SE Ridge; Tibet: N Col-NE Ridge, N Col-N Ridge, N Col-NE Ridge)]. Other seasons and other routes are infrequently attempted as they are relatively difficult and dangerous [6, 7, 12]. Finally, we excluded all records (261) for 2014, when the Nepalese side was closed after an avalanche killed 16 high-altitude porters, and for 2015 (270 records), when the mountain was again closed after an earthquake. The remaining sample included 5,324 climbers (1,916 for 1990–2005; 3,408 for 2006–2019). Age and sex were known for all individuals.

To analyze whether cause of member deaths differed by sex or by age, we collapsed “death group” assignments of HD into four groups: (1) illness or non-traumas (Acute Mountain Sickness, exhaustion, exposure/frostbite, other illness (non-Acute Mountain Sickness)), (2) falling (fall, crevasse), (3) ‘objective hazard’ (avalanche, falling rock/ice, Icefall collapse), or (4) other (Disappearance (unexplained), other, unknown). [Note: alternative categories were used by [6].] We reclassified two deaths (Z. Miletic, M. P. Maslarova) from “other” to “illness,” based on expedition and member notes in HD. To analyze whether the probability of different causes changed with age, we excluded the remaining “other” deaths (as uninformative) and used multinomial regression [13]. However, inclusion of “other” deaths did not affect the patterns (analyses not shown).

### Statistical methods

Our primary goals are to determine not only the overall rates of success, complete success, and death on Everest, but also to evaluate whether those rates have changed over time as well as whether those rates appear influenced by key socio-demographic covariates [age, sex, host country, and prior experience (i.e., whether climbers had previously attempted a Nepalese peak other than Everest)]. The first goal is straightforward, but the latter ones are not. Our prior analysis [11] used a generalized additive model (GAM) [14] to evaluate whether success or death rates varied with the covariates. We again use a GAM model for the new data (2006–2019), but add a separate dynamic coefficient model that can evaluate whether covariate effects have changed over time (see below). These two approaches are complementary and enable us to determine overall patterns and changes over time.

The prior GAM model [11] included a climber’s age via a joint-point regression [15] because an initial exploration revealed that success rates appeared to start dropping above age 40: a GAM later confirmed this “breakpoint.” When we combined the existing pre-2006 data with our newer data, that same breakpoint was still evident in success rates; and we again included age via joint-point in the new analyses. A GAM was also used to analyze age dependence of death rates, but here no joint-point was evident or used. Model fitting was performed using the “mgcv” package [16].

While performing the semi-parametric GAM analysis, we noted that the effect of year on success was discontinuous and sometimes varied wildly from year to year, likely reflecting differences in weather and conditions. Because treating year as a continuous variable was thus inappropriate, we developed another way to evaluate how effects of other covariates changed across years. This Dynamic Coefficient Model (DCM) initially separates climb data by year, estimates the model separately for each year, and then examines whether the estimated coefficients (above) show temporal trends. For example, with modern methodological advancements, is experience as beneficial to success as it used to be?

The DCM approach can evaluate temporal patterns but has limitations. First, analyzing data by year rather than by period (early vs. recent) reduces sample sizes and thus increases standard errors. Second, the resulting coefficient curves are obtained via local linear smoothing, which is sensitive to extreme points at the boundaries [17]. Consequently, we focus on interpreting the general shape of the interior part of the curve (i.e., the mid-range from 1995–2015). Note that the sign and trend of the curves are more meaningful than the specific values of the coefficients themselves, as a coefficient’s magnitude changes with measurement scale. For example, age coefficients would change nominally if we measured climber age in days as opposed to years, but the sign and trend of the curves would remain consistent.

To implement the DCM, we first have to address some DCM-specific issues. We excluded 1996 (the year of the “Into Thin Air” disaster), which was a clear anomaly. Additionally, years 1991, 1992, 1994, and 2008 were excluded because these years either had all men climbers or had all climbers using only Nepalese routes, making model fitting impossible. As noted earlier, we also excluded 2014 and 2015. Finally, we use Pearson’s chi-squared test the effects of crowding on success and death rates in recent years. Statistical modeling was performed in R (R Core Team 2019).

## Results and discussion

### Shifts in sex and in age structure

From 1953–1989, most climbers (“members”) were men: only 4.5% were by women (Table 1). However, the percentage of attempts by women has increased significantly (P < 0.001) and reached 9.1% for 1990–2005 and climbed to 14.6% for 2006–2019. Although men continue to outnumber women on other Nepalese peaks, sex discrepancies there are also slowly eroding [chart C11a in 7].

**Table 1:** Temporal trends in demographic makeup of mountaineers.

	Women		All climbers				
Time period	Mean N per year	Percentage women	Total N per year	N age ≥40 years	Percentage age ≥ 40	N age ≥60 years	Percentage age ≥ 60
**1953–1989**	2.5	4.5	2012	9.8	18.0	0.1	0.1
**1990–2005**	24.8	9.1	4332	104.9	38.7	6.1	2.2
**2006–2019**	52.7	14.6	5401	194.7	54.1	16.7	4.6

Most climbers in the early period were young (82.0% < 40 years), and few were either mid-age or old (17.9% for age = 40–59, 0.1% for ≥ 60). The percentages of attempts by mid-aged climbers and by old climbers has increased significantly (p = 2.2 x10^-16^ and 1.3 x10^-5^, respectively) and reached 36.5% and 2.2%, respectively, for 1990–2005 and then 49.5% and 4.6%, respectively, for the recent period. Thus, more than half of recent climbers are middle-aged or old (i.e., ≥ 40).

Climber diversity on Everest—by sex or by age—has clearly increased over time (Table 1), continuing a trend noted previously [11]. Comparable trends are evident in analyses of climbers on other Nepalese and border peaks [7], as well as on Denali, Alaska (6194 m) [18]. Nevertheless, gender percentages on high peaks are still far from parity [10].

Retrospective data do not permit a causal analysis of these historical shifts, which are confounded by many factors [6, 10]. Even so, the shift in sex ratio on Everest and other high peaks [7, 18] likely mirrors the increased participation by women in many sports in recent decades [19]. The broadening of age structure may be occurring because many contemporary old individuals are healthier and more active than in the past [20], and perhaps because old individuals may better able to afford the financial and time commitments of a Himalayan expedition [7, 21].

### Shifts in overall rates of success and of complete success

**Fig 1:**
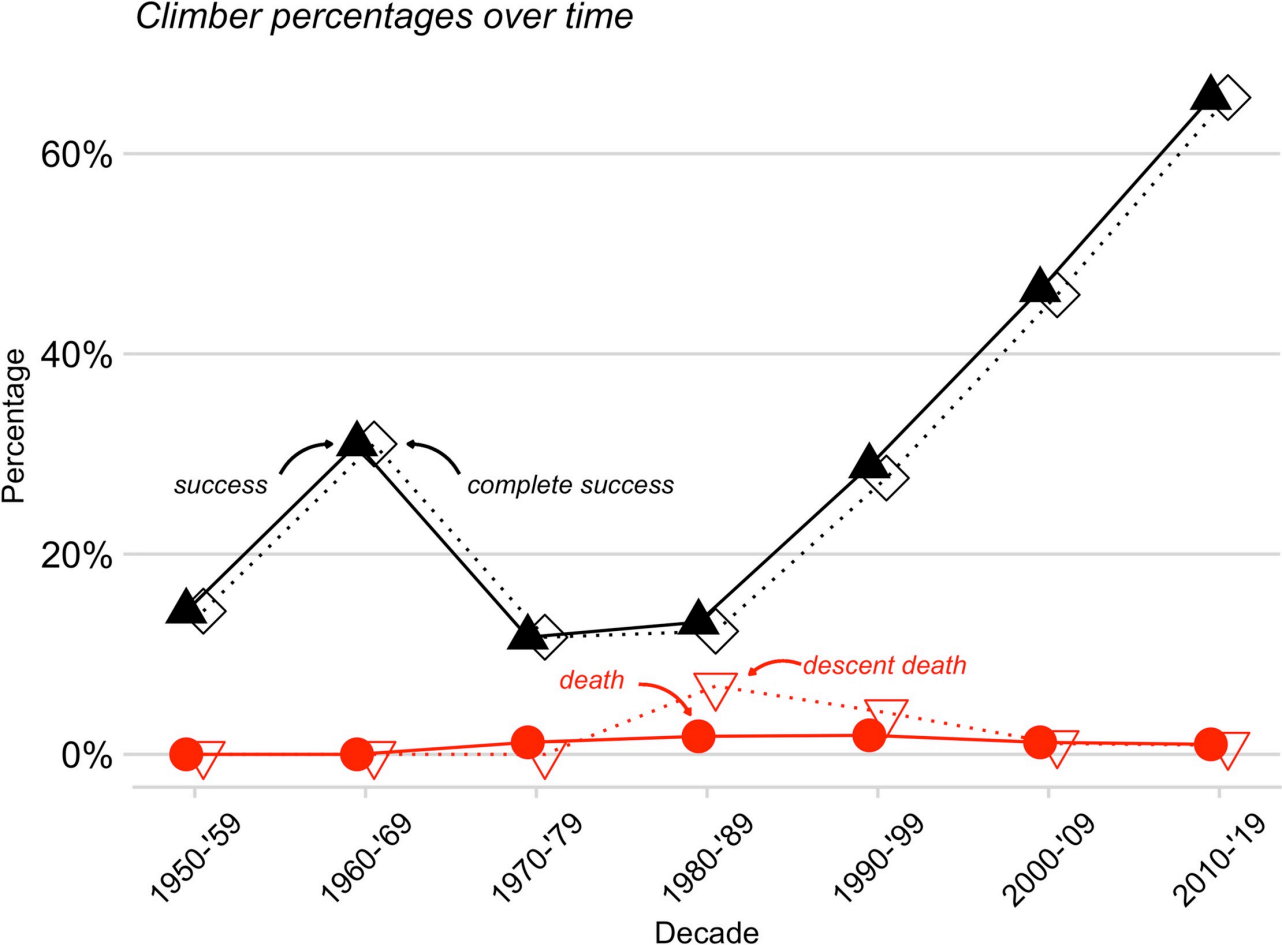
Rates of success (summited), complete success (summited and survived), overall death, and death descending from the summit. Rates are dodged to facilitate comparisons.

The overall rates of success (summiting) and of complete success (summiting and returning to base camp alive) have jumped dramatically in the past two decades (Fig 1). In fact, the success rates for 2006–2019 are essentially double those for 1990–2005 (Table 2).

**Table 2:** Rates of success, complete success, and death (as percentage) on Everest for the recent two time periods.

Year	Success (%)		Complete success (%)		All death (%)		Descent death (%)	
	Women	Men	Women	Men	Women	Men	Women	Men
**1990–2005**	32.7	32.9	31.3	32.2	1.9	1.7	1.4	0.7
**2006–2019**	68.2	64.4	68.1	63.9	0.5	1.1	0.1	0.7
*p* values	<0.001	<0.001	<0.001	<0.001	*=* 0.19 ^ns^	= 0.14 ^ns^	*NC* ^a^	*=* .99 ^ns^

Sample sizes for 1990–2005 are 214 women and 1702 men, and for 2006–2019 are 548 women and 2860 men.

^a^ The test for women’s descent death was not conducted (*NC*) due to very small counts (3/214 for 1990–2005, 1/548 for 2006–2019).

Several factors are likely involved in the marked jump in success rate (Fig 1, Table 2). Importantly, the goals of individual climbers have changed over time. During the ‘expeditionary’ period of climbing 1952–1969, 7, p. 5], expeditions were large and designed to assist a few climbers to summit. Consequently, many members had supporting roles and thus had little or no chance to summit. But in recent periods, most expeditions have been small, and most individuals are attempting to summit. For these reasons, success rates should be elevated after ~1990.

A shift in goals does not, however, explain the markedly increased success rates since 1990–2005 (Fig 1, Table 2). That recent increase likely reflects better weather forecasting, presence of fixed ropes on much of the route, accumulated logistic and route experience, improved oxygen equipment, and shifts in oxygen use [see below, 12]. Also, many contemporary expeditions are commercial [8]; and the accumulated experience and expertise of commercial companies may well enhance success rates of all climbers, even those not on a commercial expedition. Potentially, the relative number and experience of high-altitude porters assisting climbers may be more than in the past (some porters participate in many expeditions), also enhancing client success. On the other hand, contemporary crowding high on Everest (below) might well reduce individual success rates (but see below).

### Sex and success rates

In our prior analysis [11], women and men had nearly identical rates of success and of complete success. That patterns holds for the recent census (Table 2) and is further supported by a test pairing success rates of women vs. men by year (Wilcoxon signed-rank test, *p* = 0.9168, two sided).

The similarity of success rates for women and men has also been documented in a pooled survey of all Nepalese peaks for 1990–2009 [table A-30 in 7]. However, men have higher success rates than do women on some peaks in Nepal and elsewhere [7, 18], and men outperform women in shared Olympic sports [22]. Men and women do have different morphological and physiological capacities [22], but those differences to not seem to affect success rates on men and women on Everest, at least on commercial routes. Some evidence suggests that men and women may have similar physiological capacities and resistances in cold and hypoxia [23–27].

### Age and success rates

**Fig 2:**
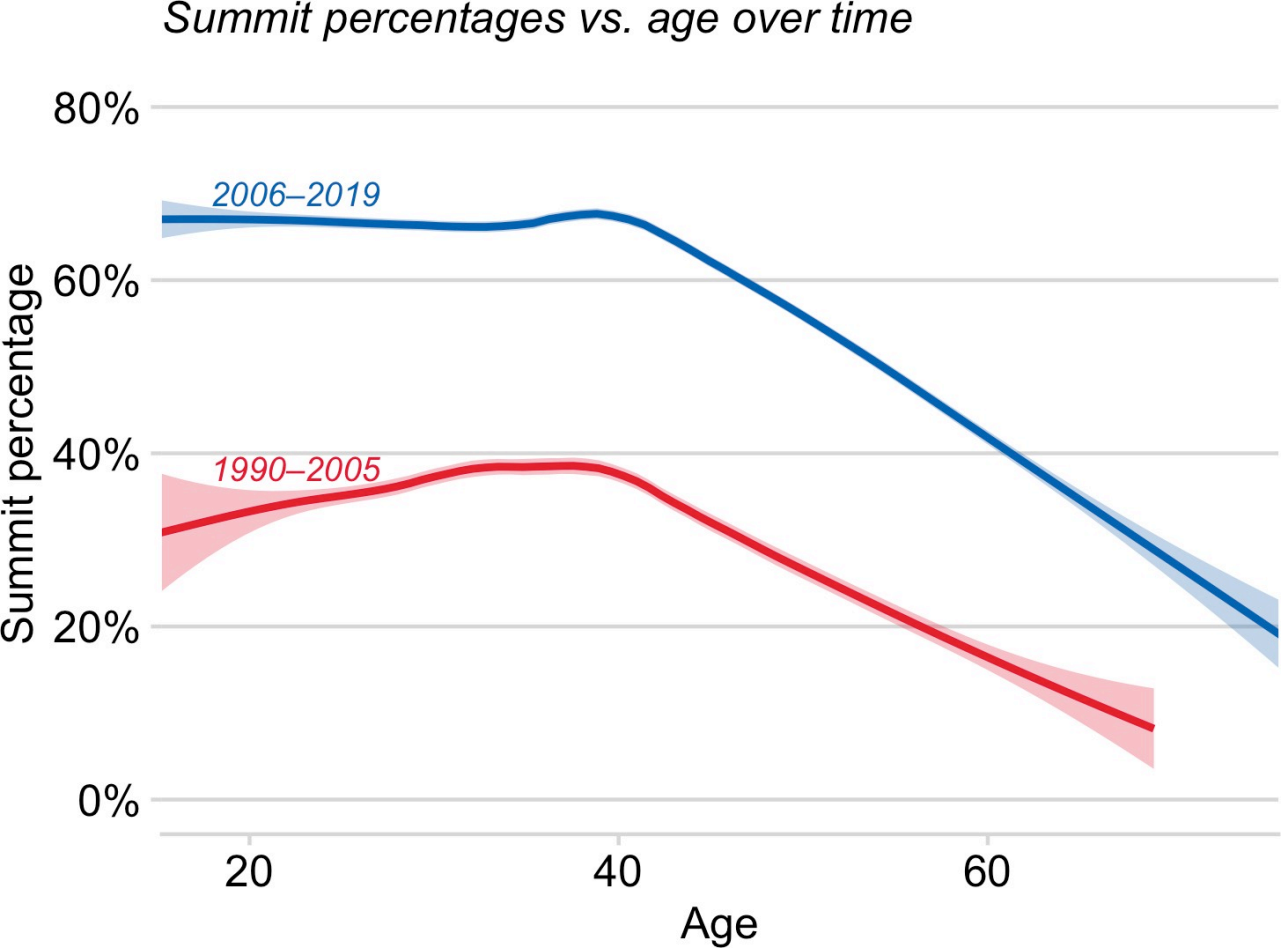
Fitted probabilities (GAM) of summiting vs. climber age for early (1990–2005, red) and recent (2006–2019, blue) cohorts. Probabilities of summiting declined for climbers older than ~ 40, but recent climbers of all ages had greatly elevated probabilities of summiting relative to early climbers. Solid lines are from local linear smoothing, and the adjacent shading represents the 95% confidence region for the expected summit probability as a function of age.

In our analysis for 1990–2005 [11], success rate was essentially flat with age until around age 40 and then declined progressively (red line in Fig 2). This qualitative pattern holds for 2006–2019 (blue in Fig 2): success rate of older climbers again declined with age after 40 (by 1.1%/year, p ≈ 0). Importantly, recent climbers older than 59 summited only about half as often as did younger climbers (33.3% vs. 63.7%). Nevertheless, climbers of all ages show a marked increase in success rate since the earlier period (1990–2005) (Fig 2). In fact, the marginal rate of summiting has essentially doubled for climbers younger than 40 (36.0% to 69.1%, p ≈ 0) as well as for those 40 and older (27.6% to 57.4%, p ≈ 0).

The age-dependence of complete success rates (summited and survived) parallels that of success rate. In both time periods, complete success rates of climbers older than about 39 declined with age. Even so, recent–both young and old–climbers had higher marginal rates than did prior climbers of comparable ages (under age 40: 68.7% vs. 35.2%, p < 2.2x10^-16^; age 40 and above: 55.7% vs. 25.6%, p < 2.2x10^-16^), when data are standardized by age group.

The decline in success rate of old climbers in the recent sample is qualitatively consistent with previous findings for 1990–2005 on Everest [11] and generally with data from other Nepalese and border peaks [7]. Similarly, old climbers have relatively lower success rates on Denali (6194 m) [18] and suggestively so on Kilimanjaro (5895 m) [28]. Old climbers may have relatively low success rates either for physical reasons [e.g., they may have reduced physiological capacities, 29] or for behavioral ones [e.g., they might be more risk averse) 11]. Note that very old climbers from Japan much higher success rates than do comparably aged climbers from other countries [see 7, p. 94].

### Experience, host country and success rates

Prior high-elevation experience may enhance the probability of summiting [9, 11]. HD records only whether individuals had attempted another Nepalese or border peak prior to attempting Everest but not whether they had attempted high peaks elsewhere (e.g., Karakorum, Alaska, Andes). Even so, climbers with experience in Nepal had higher success rates than those without experience in the current (67.6% vs. 61.5%, p ≈ 0) as well as the prior (40.9% vs. 28.1%, p ≈ 0) time periods [11].

This pattern might reflect direct benefits of experience [8, 9, 11, 30]. Alternatively, it might merely reflect self-selection: that is, climbers who first failed on a lower peak might then be less inclined to attempt Everest than would a climber who had succeeded on a lower peak [11]. In contrast, climbers who had no experience on lower peaks before attempting Everest would not have had an opportunity to self-screen and would thus include some who might not do well high altitude.

Might the enhanced success rate for recent climbers (Fig 2) reflect a potentially greater experience of recent climbers, relative to prior ones? Apparently not, as recent climbers (2006–2018) actually had significantly less experience than did earlier climbers (30.7% (recent) vs. 37.7% (1990–2005), 𝝌2 = 17.78, p ≈ 0). More likely, experience may matter less now than previously (see the ‘dynamic correlation model’ below).

Recent climbers are somewhat more likely to succeed when climbing in Nepal than in China (65.8% vs. 58.4%, *p* ≈ 0), consistent with a prior analysis [12]. This result was supported by the GAM, which returned a highly significant host coefficient (*p* ≈ 0), but not by a test that paired success rates by year (Wilcoxon signed-rank test, *p* = 0.1234). Note that the host coefficient was not significant (*p* = 0.28) in the GAM for 1990–2005 [11], suggesting that covariate effects have changed over time. We return to this issue in the context of our Dynamic Correlation Model below.

### Success: Across-year patterns (Dynamic Correlation Model)

**Fig 3:**
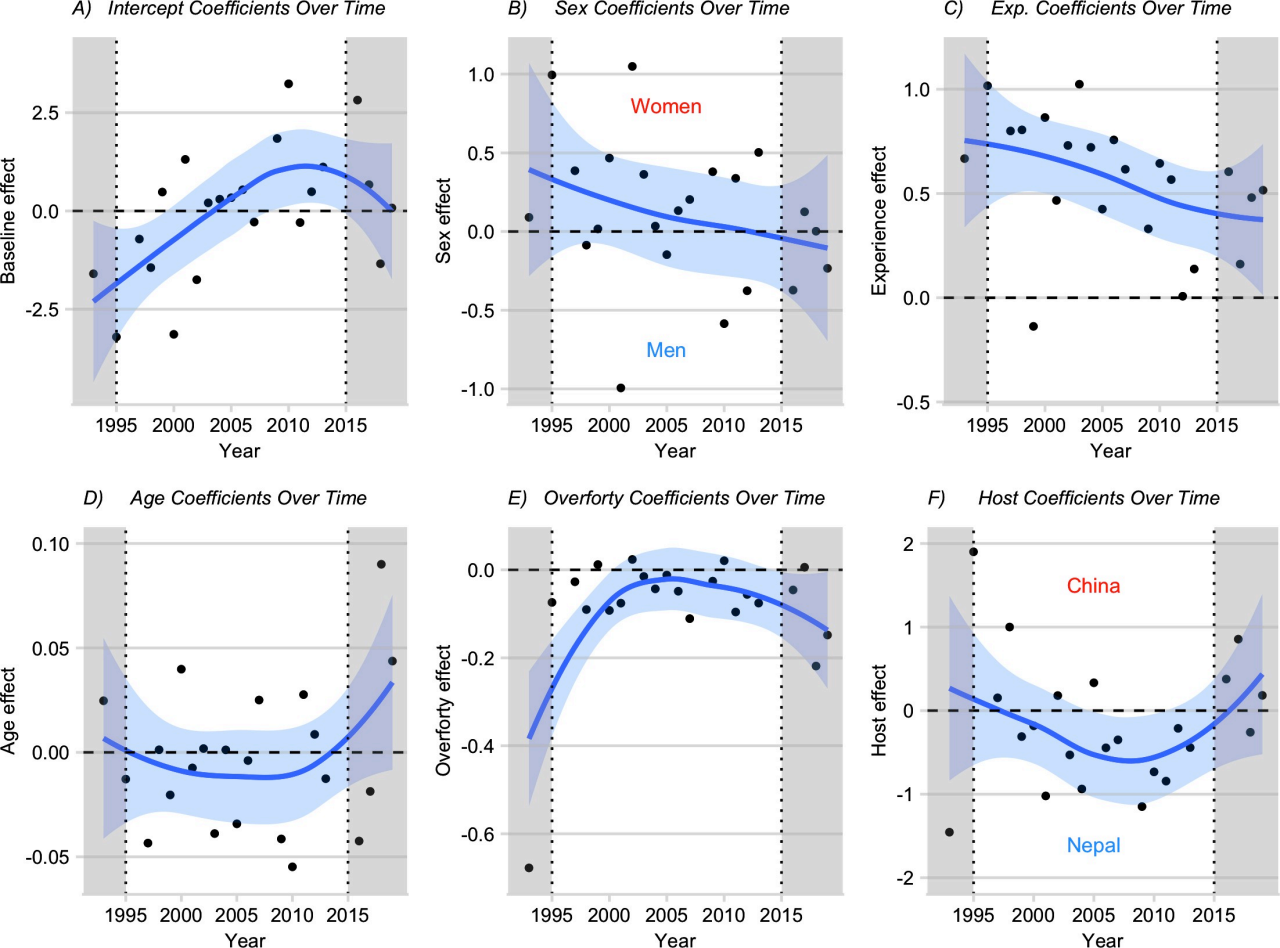
Effects of covariates (DCM) on summiting rates over time. Blue lines are obtained via local linear smoothing. Trends are reliable only in the central region (1995–2015, see Methods). The blue shaded areas represent 95% pointwise confidence regions for the mean effects as a function of year. For factor covariates (e.g., sex), a point on the “Women” side of the horizontal line indicates that women were more likely than men to summit in that year, and vice versa for a point on the “Men” side. Grayed zones indicate years which are unreliable because of boundary effects (**Methods**).

Because success rates varied noticeably among years (Fig 1), we developed a novel Dynamic Correlation Model, which examines whether covariates have consistent effects on success among years (see Methods). Results are depicted as yearly coefficient values and curves smoothed over years (Fig 3). Positive coefficients indicate that the covariate and the rate of summiting were positively related that year.

How likely a “generic” climber is to summit in a given year is indicated by each year’s intercept coefficient (Fig 3A). The overall slope of the intercept versus year curve is positive, indicating that climber success rate increased over time, consistent with the GAM analysis (above).

A positive sex coefficient (Fig 3B) would indicate that women were more likely to summit than men in a given year. However, the sex curve hovers around zero, implying that women and men had similar success rates across all years, consistent with the GAM analysis (above). The similar success rates of women and men is further supported because the confidence region fully encases the null line (dashed).

The experience curve (Fig 3C) is positive (thus experience enhances success rate) but is decreasing, implying that the positive effect of experience has declined over time. For example, positive effect of experience on summit success in 2018 (~ 0.4) was roughly one half of what it was 25 years ago (~ 0.8).

The coefficients of age (Fig 3D) and of excess age over 40 (the “overforty” variable, which is the age of climbers over 40 minus 40, Fig 3E) are predominantly negative (barring a right boundary effect on the age curve), reflecting the lower rates of summiting for older climbers, as found in the GAM analysis. When boundary years are ignored, the age and “overforty” curves are both relatively constant, suggesting age has consistently negative effects on success rate, regardless of the conditions on Everest in a given year.

A positive host coefficient (Fig 3F) would indicate (arbitrarily) that success rate was higher on a Chinese route than on a Nepalese one in a given year. However, the interior of the coefficient curve for host (China vs. Nepal) is mostly negative, indicating that between 2000 and 2015, climbers on the Nepalese side had higher rates of summiting than did those in China. After 2015, however, the curve becomes slightly positive, which suggests either that the host effect could be tilting in China’s favor or the boundary volatility is involved (see Methods).

### Death rates

Overall death rates have declined between time periods (Fig 1; Tables 2 and 3, *p* = 0.0456), though the shift is small (early 1.6% vs. recent 1.0%). Overall death rates in the recent period are similar for men and women (Table 2), are independent of experience (*p* > 0.3 for both genders), but are marginally higher on Chinese routes than on Nepalese routes (1.5% vs. 0.7%, *p* = 0.054). These patterns are qualitatively similar to those for 1990–2005 [11]. The independence of death rate from experience in Nepal is consistent with a survey of all Nepalese peaks for 1970–2010 [8] and of an analysis of elite climbers on 8000-m peaks [9].

**Table 3:** Death rates (overall and on descent from summit), numbers of deaths, and numbers of climbers at risk (boldfaced).

	Time period					
Rate	1990–1994	1995–1999	2000–2004	2005–2009	2010–2014	2015–2019
**Overall death rate**	0.8	2.4	1.3	1.3	1.1	0.8
**Total deaths**	2	12	12	17	13	10
**N climbers at risk**	**227**	**499**	**900**	**1363**	**1154**	**1181**
**Descent death rate**	0.0	6.1	1.2	1.1	1.1	0.9
**Total descent deaths**	0	9	4	8	8	7
**N summiters**	**62**	**147**	**326**	**724**	**715**	**816**

Overall death rates are for all climbers who went above base camp, and descent death rate is only for climbers descending from the summit. Numbers here reflect our primary subset of members (i.e., first-time climbers, on commercial route, in spring during non-disaster years).

**Fig 4:**
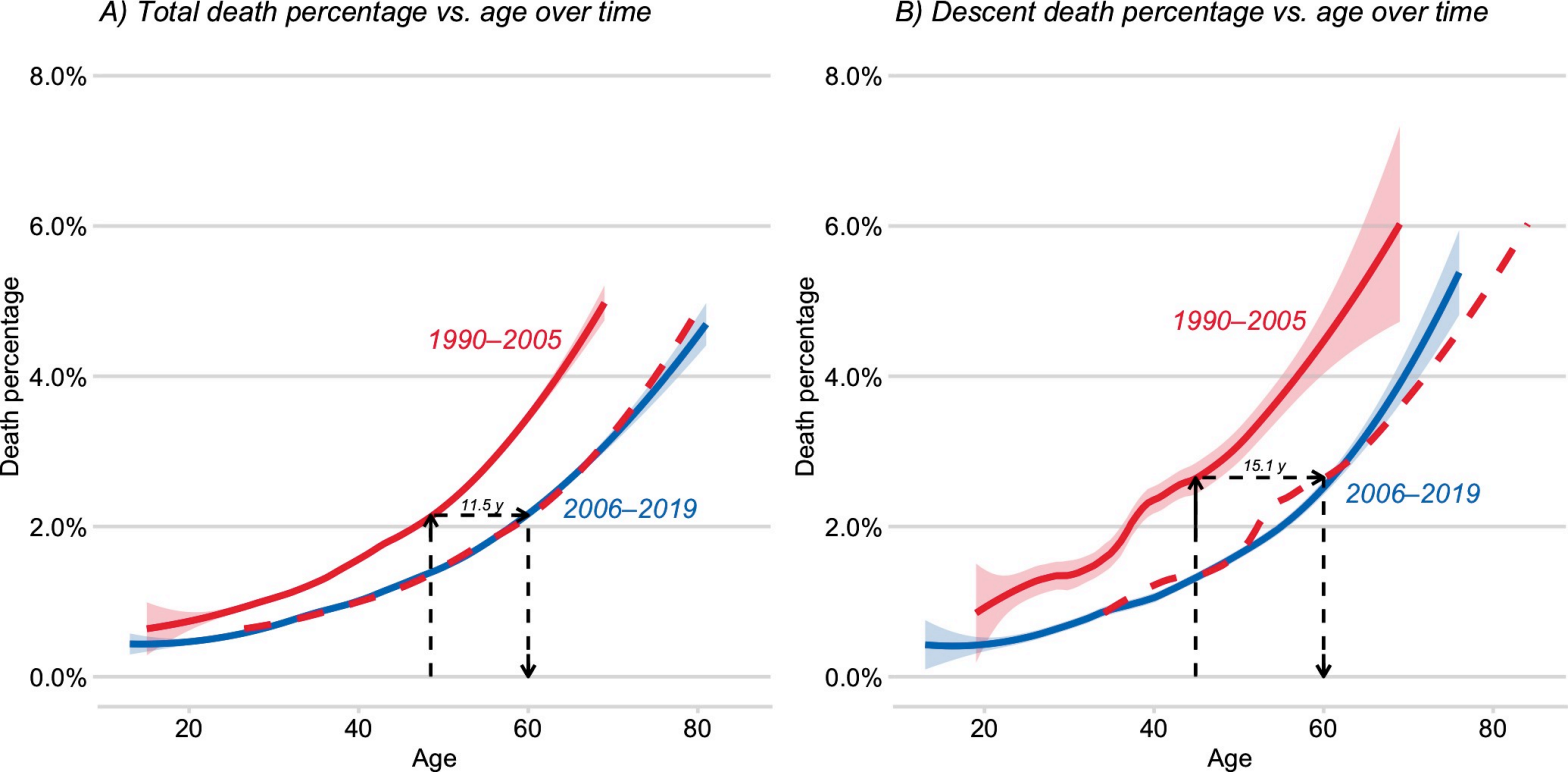
(A) Fitted probabilities of rate of death and (B) of death on descent from summit versus climber age for two time periods (red = early, blue = recent). For both periods, death rates increase with age both for all climbers who went above base camp (A) and for those who died descending from the summit (B). However, because curves for prior and recent periods appear similar in shape but differ in horizontal position (red dashed line is a right shift of the prior curve, see text), old climbers in the recent period have lower death rates than did comparably aged climbers in the prior period. In other words, the adverse effects of old age for recent climbers are delayed by about 11.5 years (A) and about 15 years (B), respectively.

Overall death rates increased with age in the recent period (Fig 4A) (GAM, *p* = 0.0003), as in the previous cohort. For example, climbers over 59 had significantly higher death rates than did younger climbers (4.1% vs. 0.9% for younger climbers, p ≈ 0, risk ratio = 4.72, 95% CI: 1.98–11.22), consistent with the patterns for 1990–2005 [11], but based on a much larger cohort of old climbers (148 vs. 52) and thus with enhanced statistical power. The death probability curves for the early and recent samples share a similar shape, differing mainly by a horizontal shift (Fig 4A). A natural estimate for this shift is the one that best aligns the two curves according to their L^2^ distance [31]. A grid search over a range of shifts suggests that the adverse effects of old age appear to be postponed by around 11.5 years for recent climbers. For example, a generic 60-year-old climber in the recent sample has roughly the same death probability as a generic 48.5-year-old in the early sample. This shift is slightly more pronounced (to the tune of one additional year) for older climbers: a generic 75 year old on a recent climb has around the same death probability as a generic 62.3 year old in an earlier climb.

Most deaths on Everest occur high on the mountain [6], and death rates of climbers descending from Himalayan summits are often high [7]. For recent Everest climbers, 61.7% of all deaths occurred after summiting, even though the time spent in descending is a small fraction of the total time on the mountain [7]. In the prior sample, only 46.9% of all deaths occurred after summiting, though the increased proportion in descent deaths is not significant from the new sample (*p* = 0.3336). Descent death rate–like overall death rate–has not shifted much over time since the 1990s (Table 3), but the adverse effects of old age are now postponed by about 15 years (Fig 4B).

**Fig 5:**
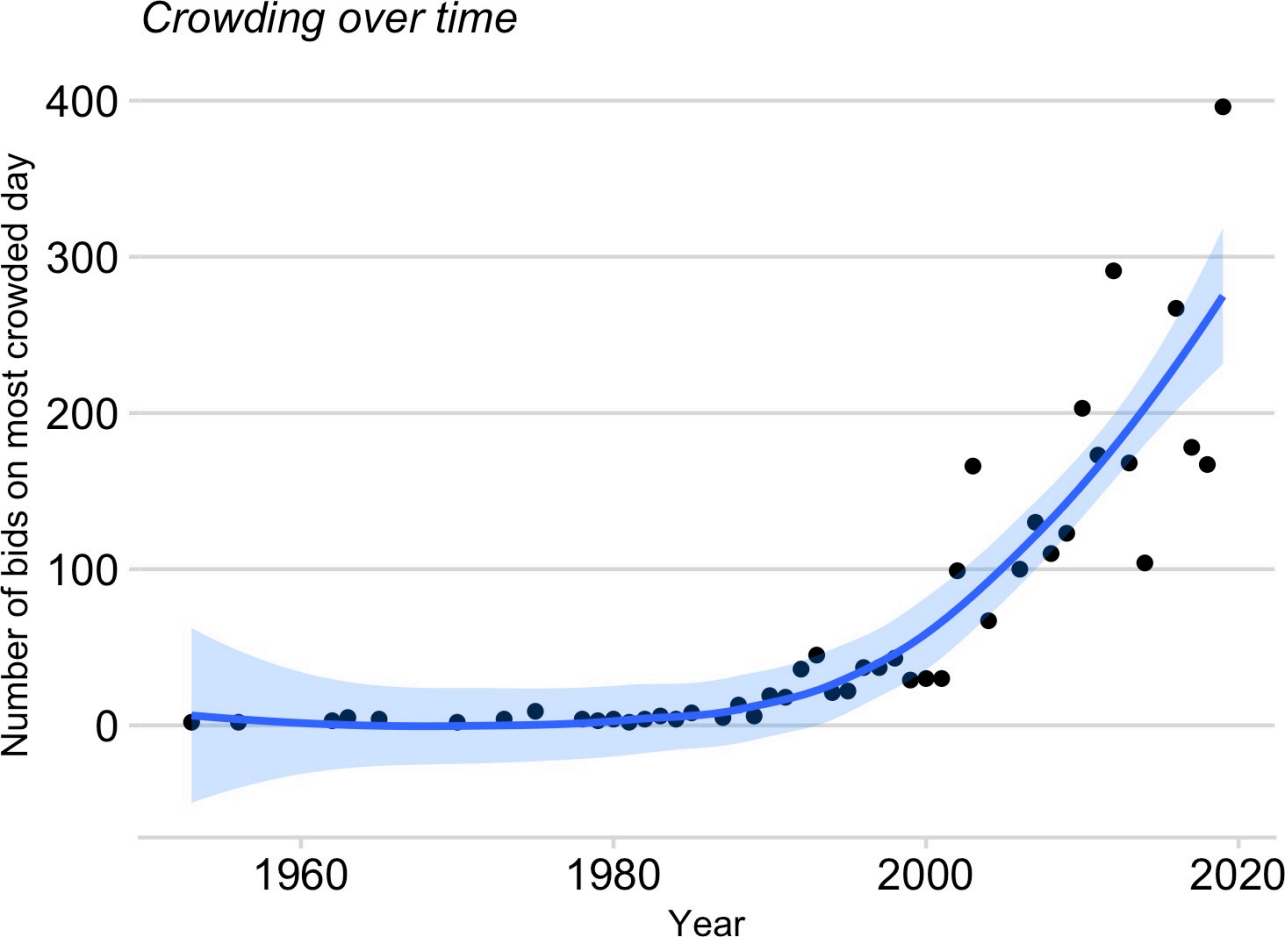
Crowding near the summit is increasing steeply in recent years. The yearly crowding index is the number of climbers making summit bids on the most crowded day of each year.

Because relatively few older climbers reach the summit (Fig 2, above), where most deaths occur, a comparison of overall death rates of young versus old (e.g., Fig 4) likely underestimates risks facing those older climbers who do summit [11]. Consequently, we performed a separate risk analyses consisting only of climbers who summitted (*n* = 2790) and thus were exposed to the same risk. In a GAM analysis conditioned on successful summiting, descent death rates were independent of sex (men 1.4%, women 0.9%, *p* > 0.8) and of experience (*p* = 0.09), but were marginally higher in China (China 2.0%, Nepal 0.9%, *p* = 0.035). As with overall death rates (Fig 4), descent death rates increased gradually with age (Fig 5, *p* = 0.005). Importantly, climbers over 59 had much higher death rates during descent than did younger climbers (10.5% vs 1.1%, *p* ≈ 0; risk ratio 10.4, 95% CI: 4.15–22.13), as in the prior time period [11]. These findings are inconsistent with a proposal that old climbers (specifically sexagenarians using supplemental oxygen) can safely climb 8000-m peaks [30].

## Age and causes of death

Causes of death on Everest have been quantified [6], but not by sex or age. Between 1990 and 2019, 119 total members (i.e., not limited to first-time climbers) died on Everest in spring. Of these, cause of death (illness, fall, or “objective hazard” such as avalanche or rock fall) could be assigned for 111 deaths (**Methods**). Because few women died during this period (N = 11), we did not separate women and men. Most assigned deaths were from illness and non-traumas (illness = 68.4%, falling = 26.1%, hazard = 5.4%).

We used multinomial regression [13], setting “illness” as the baseline category to evaluate whether cause of death changed with climber age. The coefficient for “falling” was negative (-1.968), suggesting that (of climbers who died from illness or from falling) older climbers were marginally more likely to die from illness (*P* = 0.0490). In effect, a generic old climber had roughly double the odds of dying from illness compared with a generic old climber that about 17 years younger. The coefficient for “hazard” was also negative (-0.623) but not significant (*P* = 0.5335), suggesting age was not a predictor of hazard versus illness deaths. However, a death classification at extreme altitude is sometimes unreliable [6]: a climber might literally die by falling but whose fall was caused by cognitive impairment or ataxia [6]. Thus, reported patterns are suggestive.

A team of medical experts [6] classified deaths of Everest members for 1921–2006 and recorded more deaths from traumas (54.0%: objective hazards plus falls) than from non-traumas (46.0%: high-altitude illness, hypothermia, sudden death, unclassified). Traumatic deaths were more common than in our survey, which suggests that frequency of death classes may have shifted over the history of climbing on Everest. In a survey of all Nepalese peaks for 1950–2009, 48.6% of classifiable deaths were from falling (including crevasses), 24.2% of deaths were from illness and non-traumas, and 27.2% were from objective hazards [7]. At the “Everest ER” (emergency room) at the Nepalese base camp, 85.3% of 3045 visits (not deaths) of mountaineers and a few trekkers (2003–2012) were for ‘medical reasons’ and only 14% were from trauma [32], but sex and age patterns were not analyzed.

### Crowding and crowding effects

The number of yearly summit attempts on Everest continues to rise, and the main routes are becoming increasingly crowded (Fig 5). In fact, the average number of climbers making summit bids above high camp has increased four-fold since our prior survey. Overcrowding on Everest has received attention from mountaineering associations, media, and the governments of Nepal and China. Here we quantify the trend of increased crowding and provide a preliminary look into its effect on success and death. We focus on climbers made a summit bid above the high camp, where the routes are narrow, constricted, and crowded, and where both ascending and descending climbers must use the same rope.

Climbers who reach high camp must weigh several factors (weather, snow conditions, avalanche risk) when deciding to make a summit bid. If unfavorable conditions persist for several days, climbers may accumulate at the high camp before making their bids together when conditions finally improve. To quantify how crowding has changed over the years, we determined the total number of climbers (members plus high-altitude porters) making a summit bid on the single most crowded day of each year. Crowding was limited until around the early 1980s, but then began to increase exponentially (Fig 5). On 23 May 2019, 396 climbers made summit bids!

Is increased crowding affecting the probabilities of success and of death? A reasonable expectation is that crowding will slow ascents and descents, increase time in the “death zone,” and thus decrease success probabilities but increase death ones. Testing these expectations is challenging and is beyond the scope of this paper. However, we provide an initial test to examine within-year correlations between rates and crowding. We focus on the two most recent years.

In 2019, climbers made summit bids on 13 days, and 65.9% were on just two days (22–23 May). We classified these two days as “crowded.” Neither the probability of summiting (92.4% on crowded days, 90.5% uncrowded) or the probability of dying (1.0% for crowded, 1.2% for uncrowded) were significantly different (both *p* ≈ 1.0).

In 2018, climbers made summit bids on 11 days, and 58.2% were on four days (16–19 May). The probability of summiting did not differ (*p* ≈1.0) between crowded (91.1%) and uncrowded days (95.3%) days. No climber making a summit bid died in 2018.

These results are contrary to the expectation that crowding high on Everest will reduce success but increase risk of death. In the two springs (2018–19) sampled here, success and death rates not distinguishable between crowded and uncrowded days. Crowding almost certainly will slow climbers, but any negative effects of crowding may be masked by the benefits waiting at high camp and then making a summit bid only when conditions appear suitable (weather, snow).

## Concluding remarks and implications

Here we present recent patterns (2006–2019) of success and of death rates on Everest as well as evaluate whether those patterns have changed from those from 1990–2005. Despite marked year-to-year variation in rates, some patterns are conspicuous and are qualitatively similar to those documented for Everest for 1990–2005 [11] and for other Nepalese peaks [7]. Quantitative differences are, however, striking. Here we highlight several key patterns and discuss their implications for climbers and for agencies that determine climbing regulations.

The probability of summiting has increased dramatically. In fact, the overall probabilities of success and of complete success in 2006–2019 are essentially double those estimated for 1990–2005 (Table 2, Fig 1). About two thirds of climbers who went above base camp reached the summit in the recent sample. Possible reasons for this shift are evaluated above.

Women are a small but increasing proportion of Everest mountaineers (Table 1), and women and men have very similar success and death rates in spring on commercial routes (Table 2), as was the case in our prior sample [11] and for Nepalese peaks in general [7]. Whether women and main have similar rates for other seasons and routes is unstudied and will require larger sample sizes than are currently available.

More older climbers are attempting Everest. In the early decades after the first ascent (1953), most climbers were younger than 40; but thereafter the age distribution has been progressively broadening on Everest [11](Table 1) as well as other Nepalese peaks [7]. Notably, the proportions ‘old’ and of ‘very old’ climbers have been increasing (Table 1). But relative to young climbers (< 40 years), these older climbers have reduced chances of summiting (Fig 2) as well as elevated chances of dying (Fig 4), especially if they had summited (Fig 4, Table 2). Even so, generic 60-year-old climbers had almost double the success rate in 2006–2019 compared to comparably aged climbers in 1990–2005 (Fig 2). Moreover, the age threshold at which risk of death increases has shifted to an older age (Fig 4); and the probability of dying from illness increases marginally with age.

These patterns are directly relevant to current debates over whether age restrictions should be placed on climbers attempting Everest (one proposed upper limit for Nepal is 75). Our data demonstrate that old age is a disadvantage to both success and death rates on Everest. Nevertheless, old age is not an absolute barrier to summiting: indeed, old climbers (≥ 60) in our recent sample have markedly higher success rates than did comparably aged climbers just a few decades ago (Fig 2). Yuichiro Miura (Japan) even summited at age 80 (his fifth time on the mountain and his third summit on Everest), though he needed to be air lifted down from a high camp (records in HD). Min Bahadur Sherchan (Nepal) was 85 when he attempted to break Miura’s record, but died in base camp. In any case, because only 0.07% of climbers (n = 3) in the past decade were older than 75, any imposed restriction to younger ages will have little impact on crowding or on overall deaths.

Mountaineering data are descriptive and retrospective, and thus confounds might bias patterns. For that reason, we analyzed the impacts of key socio-demographic variables (age, gender, climbing route, year, and prior high-altitude experience) that are known influence rates on Everest [5, 7, 11]. Other factors could influence these rates. For example, supplemental oxygen seemingly affects success and death rates [5, 7]. We did not analyze it here because more than 95% of all summit attempts involve supplemental oxygen [33]. Whether changes in use of supplemental oxygen might have helped elevate success rates is unknown: a few climbers are starting to use supplemental oxygen lower on the mountain, and some are using elevated flow rates (Eric Simonson, personal communication). Team size and relative number of high-altitude assistants may be important, but contemporary teams merge and climb together on main routes and are thus not independent. Commercial versus non-commercial expeditions had similar death rates [8], and both types also merge on the same routes. Other confounds (e.g., team leadership, weather, nationality, genetics, use and type of medications, ozone exposure) could potentially be examined in future studies [7, 28, 34–36].

Finally, the purported inexperience of many contemporary Everest climbers has received much publicity in the media, raising concerns that inexperience will increase death rates. The Nepalese parliament recently postponed a decision about restricting permits only to those who have summited at least one Nepalese peak higher than 6500 m. Our analyses confirm that climbers in 2005–2019 had less high-altitude experience (in Nepal) than did those climbing in 1990–2005. However, the effect of prior experience on success rates is small and has declined in recent years, possibly because of increased reliance on commercial expeditions. More importantly and somewhat surprisingly, experience was unrelated to death rates in our current and prior samples, in a comprehensive survey of Nepalese peaks [8], and also in a detailed analysis of elite mountaineers [9]. Implementation of this proposed restriction would substantially reduce the number of climbers on Everest: 69.6% of climbers who received permits issued by Nepal (2015–2019) had not previously summited a Nepalese peak. This restriction would also reduce foreign income to Nepal and to all those who support–and are supported by–Everest mountaineers.
